# False positive elevation in serum creatinine: a case report

**DOI:** 10.3389/fmed.2024.1375173

**Published:** 2024-03-04

**Authors:** Laia Oliveras, Ana Coloma, Teresa Escartín, Maria José Castro, Natalia Vicente, Montse Gomà, Josep Maria Cruzado

**Affiliations:** ^1^Department of Nephrology, Hospital Universitari de Bellvitge, Institut d’Investigació Biomèdica de Bellvitge (IDIBELL), L’Hospitalet de Llobregat, Spain; ^2^Clinical Laboratory, Hospital Universitari de Bellvitge, L’Hospitalet de Llobregat, Spain; ^3^Department of Pathology, Hospital Universitari de Bellvitge, L’Hospitalet de Llobregat, Spain

**Keywords:** IgM, monoclonal gammopathy, spurious creatinine, falsely elevated creatinine, case report

## Abstract

**Background:**

Paraproteins can interfere with several substances, producing erroneous laboratory measurements. The diagnosis of kidney disease in patients with hematological disorders has important prognosis implications. An elevated creatinine with no other signs of kidney disease should prompt the idea of a spurious creatinine. Communication between the clinical team and the laboratory is key.

**Case presentation:**

In this case, we present a 68-year-old woman with an elevated creatinine and an IgM lambda paraprotein. Interestingly, there were no other signs of chronic kidney disease besides the creatinine value, with no albuminuria or microhematuria. A kidney biopsy showed normal parenchyma and ruled out the possibility of paraprotein-related damage. The monoclonal component and creatinine levels raised parallelly during follow-up while maintaining normal urea levels. This prompted the hypothesis of a falsely elevated creatinine. It was confirmed with a normal glomerular filtration rate determined by a radioisotope, a cystatin C measurement and a reduction in creatinine when diluting the sample.

**Conclusion:**

It is important to consider the possibility of a falsely elevated creatinine in patients with paraproteinemia and no other signs of kidney disease to avoid unnecessary diagnostic tests and for the prognostic implications.

## 1 Introduction

Paraproteins can interfere with various analysis methods by increasing turbidity or precipitation, leading to an erroneous measurement (1, 2). The Jaffe and enzymatic methods are the most common for measuring serum creatinine. Jaffe method is based on the reaction of creatinine with picric acid, and the enzymatic method on the conversion of creatinine to glycine, formaldehyde, and hydrogen peroxide by the action of creatininase, creatine kinase, and sarcosine oxidase (3).

The Jaffe method can be interfered with by several substances apart from paraproteins, such as bilirubin, glucose, acetoacetate, or various drugs (3). Although the enzymatic method is less susceptible to paraprotein interference, several cases have been reported (4, 5). Also, a recent report has described a case of negative interference from IgM paraprotein, causing a very low creatinine value (6).

## 2 Case report

A 68-year-old woman was referred to our Nephrology Department for kidney failure and a monoclonal gammopathy. Her medical history included dyslipidemia and hypothyroidism, taking statins and levothyroxine. There was no drug abuse or nephrotoxic medications. She was clinically well and did not complain of any symptoms. Physical examination was unremarkable.

Laboratory findings included a creatinine elevation to 246 μmol/L with an estimated glomerular filtration rate (eGFR) of 17 mL/min and urea of 5.1 mmol/L. Reports from previous years showed a normal kidney function. Calcium adjusted for albumin was 2.22 mmol/L, with a total protein level of 80 g/L. Phosphate, calcidiol, and parathyroid hormone levels were normal. Lipid and hepatic profiles showed no significant alterations. Creatine kinase, thyroid hormone levels, and complete blood count were normal. Immunofixation electrophoresis confirmed a serum monoclonal IgM-lambda of 2.0 g/L. There was no evidence of albuminuria or proteinuria, with negative urine immunofixation, and no microhematuria.

Abdominal ultrasound showed symmetrical kidneys, with normal corticomedullary differentiation.

**Figure 1:**
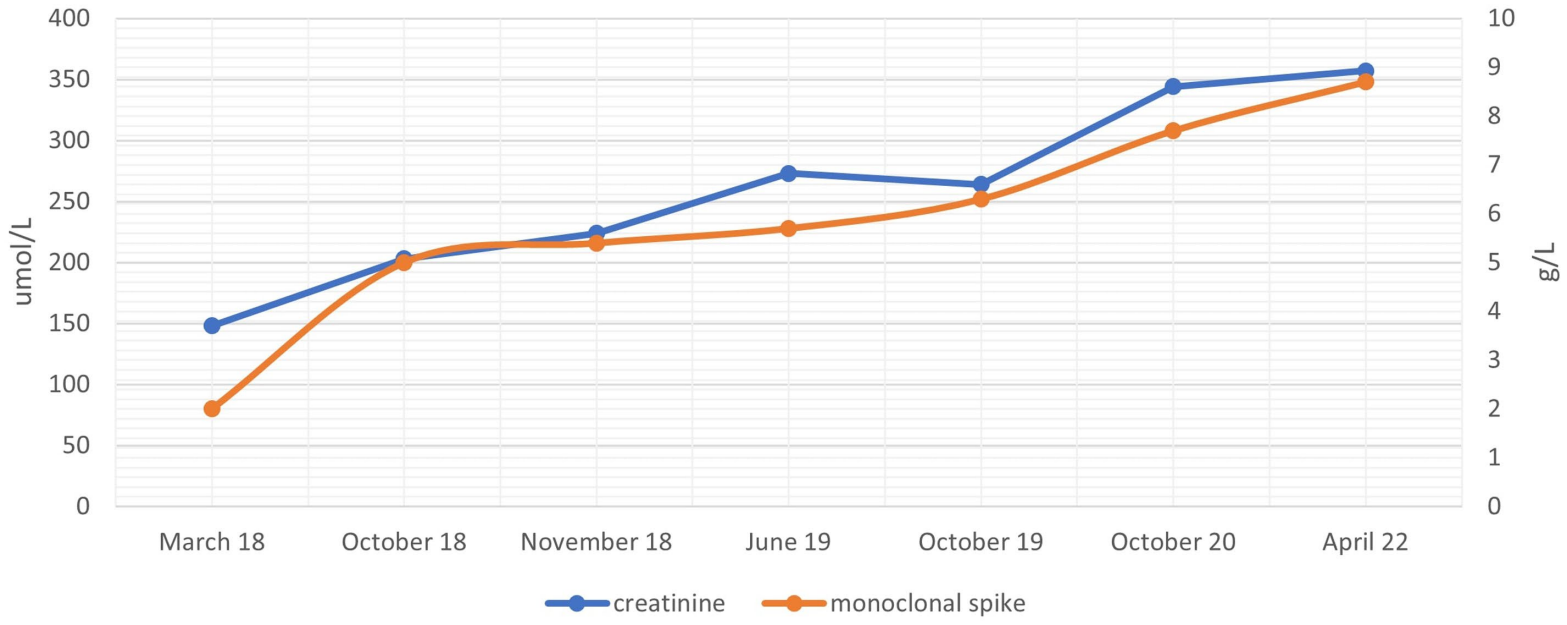
Creatinine and monoclonal IgM-lambda evolution through time.

Laboratory tests were repeated, showing a progressive increase in serum creatinine up to 347 μmol/L (eGFR 11 mL/min) and in the serum monoclonal IgM-lambda to 7.9 g/L (Figure 1). Other parameters showed no significant change over time.

**Figure 2:**
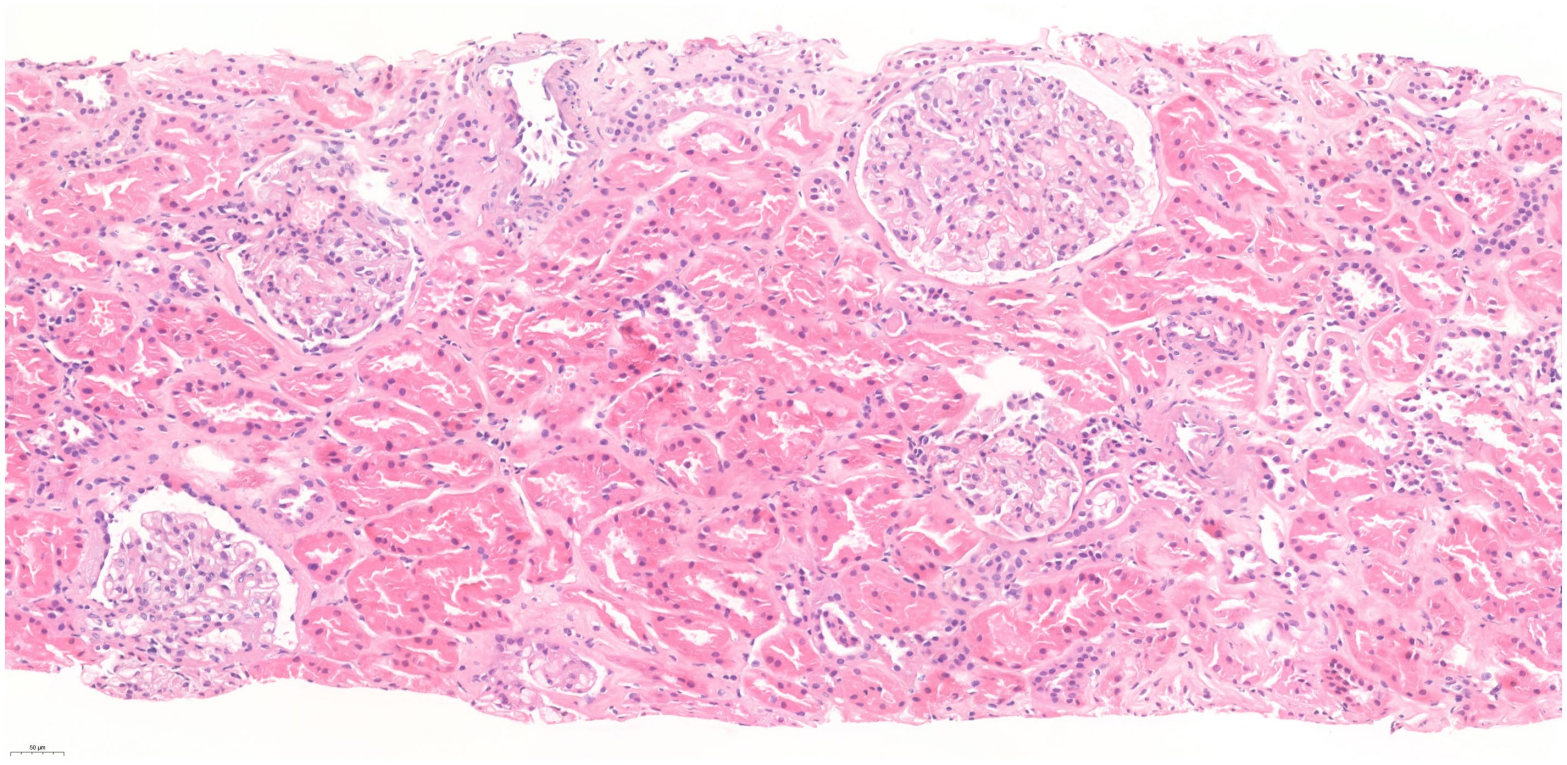
Kidney biopsy, hematoxylin–eosin stain.

Bone marrow aspiration immunophenotyping showed 4.86% B-lymphocytes with lambda light chain predominance, and Congo red stain was negative. The kidney biopsy showed a total of 16 glomeruli, one of them globally sclerosed. There was no tubulointerstitial scarring or inflammation. No alterations were observed in the cortical blood vessels (Figure 2). Immunofluorescence was negative as well as Congo red stain. Electron microscopy was not performed.

To clarify the inconsistent results of an elevated creatinine with no other signs of kidney disease, we measured the glomerular filtration rate with chromium-51 labeled ethylenediamine tetraacetic acid (51Cr-EDTA), which showed a value of 77 mL/min/1.73 m^2^.

These results prompted the idea that the elevated serum creatinine levels could be spurious and related to an interference with the paraprotein during the measurement method at the laboratory. The rise in the creatinine level over time was concordant with an increase in the monoclonal component, while normal urea levels were maintained. Our laboratory uses an enzymatic method to measure creatinine. The only available method in our laboratory to verify if the interference is due to the IgM paraprotein is to perform a dilution of the serum sample, thereby diluting the presence of the interfering element (IgM molecules) as well. The IgM concentration was 8,730 mg/L. A 1:4 dilution of the patient’s serum sample was carried out by mixing 100 μL of physiological saline with 300 μL of serum, and the creatinine concentration was measured. The obtained result was 27 μmol/L, which, when multiplied by the dilution factor, would yield a result of 108 μmol/L serum creatinine. The serum index measured in the sample was a hemolysis index of 4 μmol/L, an icteric index of 17 μmol/L, and a lipemic index of 21. None of these serum indices interfere with the measurement of serum creatinine concentration. We confirmed these results with a cystatin C measurement, with a value of 1.01 mg/dL (eGFR 70 mL/min). Our patient was finally diagnosed with a falsely elevated serum creatinine associated with an IgM paraprotein.

## 3 Discussion

To rule out a spurious creatinine, a first approach in the laboratory would be to try to minimize or eliminate the interference, by diluting the sample or pre-treating it with deproteinizing agents. Next steps would include determining other renal biomarkers such as cystatin C or measuring the glomerular filtration rate by other available methods.

Over time, creatinine value rose parallelly to the monoclonal component increase, maintaining normal urea levels. In our case, we confirmed through multiple methods that creatinine was falsely elevated. We determined the glomerular filtration rate by a radioisotope, performed a cystatin C measurement, and several dilutions at different time-points to ascertain creatinine concentration, confirming normal serum creatinine values. A kidney biopsy was performed before the diagnosis of the falsely elevated creatinine, and there were no signs of paraprotein-related kidney disease on light microscopy. We did not perform further steps such as an electron microscopy or immunofluorescence on paraffin-embedded tissue after pronase digestion because of the evidence that the creatinine was falsely elevated and that there were no clinical signs of actual clinical damage.

The exact mechanism by which only a small proportion of patients with paraproteinemia experience erroneous creatinine results has not been described. It is hypothesized that differences in the variable region of the IgM paraprotein could confer avidity for some chromogens to bind (6).

There are several published cases with IgM paraproteins interfering with creatinine measurements, but only one other involved IgM-lambda (7). To our knowledge, our case has the lowest monoclonal component (2.0 g/L, while others range from 3.6 to 44 g/L). This indicates that even minor monoclonal bands can cause interferences. Also, our case report has the longest follow-up, showing the interrelation between creatinine and the monoclonal band for up to 5 years.

Even though seldom encountered, it is important to consider the possibility of a falsely elevated creatinine in patients with paraproteinemia when serum creatinine increase is inconsistent with clinical signs, even with minor monoclonal bands. This point is particularly relevant considering the prognostic implications of kidney disease in patients with hematological diseases. In this regard, communication between the clinical team and the laboratory is paramount.

## Data availability statement

The original contributions presented in the study are included in the article/Supplementary material, further inquiries can be directed to the corresponding authors.

## Ethics statement

Written informed consent was obtained from the individual(s) for the publication of any potentially identifiable images or data included in this article.

## Author contributions

LO: Writing – original draft. AC: Writing – review & editing. TE: Writing – original draft. MC: Writing- review & editing. NV: Pathology images, Writing – review & editing. MG: Pathology images, Writing-review & editing. JC: Conceptualization, Writing – review & editing.

## References

[1] Berth M Delanghe J. Protein precipitation as a possible important pitfall in the clinical chemistry analysis of blood samples containing monoclonal immunoglobulins: 2 case reports and a review of the literature. Acta Clin Belg. (2004) 59:263–73. doi: 10.1179/acb.2004.039, PMID: 15641396

[2] Yang Y Howanitz PJ Howanitz JH Gorfajn H Wong K. Paraproteins are a common cause of interferences with automated chemistry methods. Arch Pathol Lab Med. (2008) 132:217–23. doi: 10.5858/2008-132-217-PAACCO, PMID: 18251580

[3] Ou M Song Y Li S Liu G Jia J Zhang M. LC-MS/MS method for serum creatinine: comparison with enzymatic method and Jaffe method. PLoS One. (2015) 10:e0133912. doi: 10.1371/journal.pone.0133912, PMID: 26207996 PMC4514740

[4] Salter T Marsh J Sood B Livingstone C Gallagher H. Pseudohypercreatininaemia in two patients caused by monoclonal IgM interference with enzymatic assay of creatinine. J Clin Pathol. (2015) 68:854–5. doi: 10.1136/jclinpath-2015-203064 26060264

[5] Mase H Hamano N Mizuhara R Nozaki T Saso T Wada T. Falsely elevated serum creatinine associated with IgM paraproteinemia. Kidney Int Rep. (2020) 5:377–81. doi: 10.1016/j.ekir.2019.11.017, PMID: 32154461 PMC7056855

[6] Flowers KC Tuddenham E Leiva A Garrison L Morris JE Cromwell T. Negative interference from immunoglobulin M paraproteinaemia on the Roche enzymatic creatinine method. Ann Clin Biochem. (2022) 59:205–10. doi: 10.1177/00045632221074867, PMID: 35133213

[7] Abdelkarim Metraiah EH Regan H Louw J Kidder D. Deceiving proteins! A case of lymphoma and high creatinine. BMJ Csae Rep. (2017) 2017:bcr2016217946. doi: 10.1136/bcr-2016-217946 PMC5278318 28115403

